# The Lived Experience of Ambiguous Marital Separation: A Phenomenological Study

**DOI:** 10.1111/jmft.12419

**Published:** 2019-12-13

**Authors:** Sarah A. Crabtree, Steven M. Harris

**Affiliations:** ^1^ Boston University; ^2^ University of Minnesota

## Abstract

Researchers have long treated marital separation as a linear transition that inevitably leads to divorce. Popular sources suggest that some couples separate without clarity about how the separation will end, often to assess whether to divorce or stay married. However, to date, we could not locate any empirical research on this kind of ambiguous separation. With a sample of 20 currently separated persons from around the United States, this study employed a hermeneutic phenomenological design to inquire about the experience of separating from one's spouse when the separation was initiated without clarity about how it would end. Six essential themes emerged: (a) our relationship feels ambiguous, (b) separation is a private experience, (c) separation is a lonely experience, (d) benefits to separating, (e) separation is not sustainable, and (f) the outcome is unclear. The article concludes with a discussion of and implications for the study findings.

Researchers have long been interested in marriage and divorce, but divorce has been largely treated as a single event, which oversimplifies a complex process. In recent years, some divorce researchers have used a “multiple transition perspective,” through which they examine how transitions following a divorce account for variance in outcomes (Amato, 2010, p. 657). Others have examined what precedes divorce, such as divorce ideation when a marriage is strained (Hawkins et al., 2017). These process‐ and transition‐oriented perspectives show promise in better understanding marital dissolution.

Marital separation is an under‐studied phenomenon related to divorce‐related processes and transitions. During a separation, a couple remains legally married, but their relationship is on hold, either legally or informally, due to relationship distress. Amato (2010) called separation a “socially ambiguous status—not quite married, not quite divorced” (p. 661). The larger divorce literature has rarely differentiated separated persons from those who are divorcing in study samples (e.g., Sbarra, Smith, & Mehl, 2014). While previous research suggests that divorce is most likely (e.g., Binstock & Thornton, 2003), as Weiss (1975) stated, “every divorce implies a separation, but the converse most definitely is not true” (p. 4). Estimates suggest that 6% (Vennum et al., 2014) to 18% (Kitson, 1985) of still‐married U.S. couples have separated at some point in their marriage. Treating separated persons as divorcing suggests an assumption that separation inevitably and linearly leads to divorce and leaves little room to examine different outcomes (e.g., reconciliation, long‐term separation), pathways to various outcomes, or unique elements of the separation experience. This also implies that marital decision‐making happens prior to separating when it may continue after. For example, in a sample of couples who had filed for divorce, Doherty, Willoughby, and Peterson (2011) found that both spouses in one in nine couples believed the marriage could be saved, and both partners in 1 in 10 couples expressed interest in reconciliation services, suggesting a more complex path between marriage and divorce than much of the literature implies.

A small body of literature has conceptualized and treated separation as distinct from divorce and has primarily examined outcome predictors and instability in reconciled marriages. Key findings suggest outcomes are predicated on access to resources, as those with fewer financial and social resources have been more likely to stay, or attempt to stay, in a marriage following a separation (Morgan, 1988; Tumin et al., 2015; Wineberg & McCarthy, 1994). This may be especially salient among women, who tend to think less favorably about reconciliation (Bloom & Hodges, 1981) but who are more likely to experience financial hardship following a divorce (de Vaus, Gray, Qu, & Stanton, 2017). Staying in a marriage does not mean a couple has resolved their marital problems, however. Persons who have separated but stayed in their marriages report greater instability and less happiness than those who never separated (Binstock & Thornton, 2003; Kitson, 1985; Vennum et al., 2014; Wineberg & McCarthy, 1994).

Most separation studies emerged in the 1980s and 1990s, we suspect in response to rising divorce rates in the United States and shifts in family life. No‐fault divorce laws passed around the country, the average age of marriage and number of cohabiting couples increased, and gender norms were in flux both in and outside the family (Cherlin, 2004; Knox, 1980). Much has continued to change that has affected couple relationships more broadly, such as the effects of social media (e.g., McDaniel & Coyne, 2016) or the federal legalization of same‐sex marriage and subsequently same‐sex divorce (e.g., Hoy, 2019), and which could possibly affect marital decision‐making processes. Research findings are situated in their historical contexts (Chesla, 1995), and the extent to which these dated findings are relevant today is unclear. Furthermore, we could find no qualitative studies on separation. While qualitative inquiry can be valuable at any time, Greenstein and Davis (2012) suggest researchers begin with exploration and description of an underexplored phenomenon to help guide further research. The separation literature primarily relies on secondary, quantitative datasets (e.g., Binstock & Thornton, 2003; Wineberg & McCarthy, 1994), and key variables may be missing from the analyses because either they were not available in the datasets or have not yet been identified as relevant for empirical investigation. Qualitative methodologies, specifically those that emphasize the articulation of lived experience, could help illuminate parts of separation that researchers may have missed and that might provide guidance for future investigation. Given the scarce empirical work in this area, obtaining purposeful samples of currently separated persons who can provide insight into the separation experience is imperative for advancing this body of literature.

While there is much to consider in further exploring separation and understanding its function in marital decision‐making, differentiating types of separation seems especially critical. Conceivably, many couples separate because they intend to divorce; however, these separations are conceptually different from *ambiguous separations—*those that begin without clarity about an outcome because one or both spouses is unclear about whether to stay married. Popular sources promote ambiguous separations, sometimes calling them trial, controlled, or managed separations (e.g., Lipe, 2010; Raffel, 1999), to aid in decision‐making about the future of a marriage. Researchers have commented on ambiguous separations (e.g., Tumin, Han, & Qian, 2015), but we could not locate any research on them. In fact, the empirical effects of ambiguous separation on families and its utility for marital decision‐making are largely unknown. We suspect that the absence of clarity about a separation's outcome might influence how a separation is experienced. For example, divorcing couples need to negotiate and define new relationship terms (e.g., Peterson & Hendricksen Christiansen, 2008), but ambiguously separated couples may delay these kinds of negotiations because of the possible impermanence of their decisions. Additionally, prior research has documented the negative mental health effects of uncertainty intolerance (Carleton et al., 2012), and uncertainty about a marriage's future may contribute to unique kinds of distress. However, these possibilities are largely unexplored.

## The Current Study

Separation is often conflated with divorce, and to date, we could find no empirical literature on ambiguous marital separation despite popular literature promoting it as a strategy to obtain clarity about a marriage's future. The purpose of this study is to advance the separation literature by learning about the lived experience of ambiguous separations to inform future research on and clinical practice with individuals and couples who are deciding about the future of their marriage. Utilizing qualitative methods, this study asked, “*What is the experience of being separated from a spouse when the separation is initiated without clarity about how it will end because one or both spouses is deciding whether to divorce or stay married?”*

## Methods

### Study Design

This study employed a hermeneutic phenomenology design. Phenomenology emphasizes the lived, meaning‐filled experience of a phenomenon (Giorgi, 2010; Wojnar & Swanson, 2007) and aims to “transform lived experience into a textual expression of its essence” (van Manen, 1990). Hermeneutic phenomenology assumes the interpretive nature of experience in historical contexts (Chesla, 1995), collaborative interaction between researcher and participants, and representation of the participants' narratives as interpreted by the researcher (van Manen, 1990; Wojnar & Swanson, 2007).

### Participants

The sample consisted of 20 married individuals who, at the time of the interviews, identified as separated from their spouse and whose separations began without clarity about how they would end, were 25 years of age or older, and parent to one or more child with their spouse. We recognize that a phenomenon similar to ambiguous marital separation may also occur in unmarried cohabiting couples, but we opted for more specificity in our sampling frame to avoid conflating potentially unique experiences. In all, 14 participants identified as women, and 6 as men. Their average age was 36.4 years, and they had an average of 2.45 children. The average separation length was 5.7 months excluding one participant who had been separated for 9 years; the remaining participants had been separated between 1 and 24 months. In total, 16 participants identified as white, 1 as black or African American, 1 as Hispanic/Latinx, and 2 as biracial. In all, 14 of the participants (12 women and 2 men) initiated the separation. Annual household incomes were $19,999 or less (10%), $20–39,999 (5%), $40–59,999 (20%), $60–79,999 (30%), $80–99,999 (5%), and $100,000 or more (30%). All participants were in female–male marriages.

### Recruitment

Self‐selected and snowball strategies were used to recruit the study sample. To reach participants from various backgrounds and geographic regions, and considering that separated persons may be difficult to locate with an ambiguous marital status, sponsored Facebook advertisements targeted United States users who (a) had listed a relationship status of “married” or “separated” and (b) were 25 years of age or older. The advertisements linked to a Facebook page where potential participants could read more about the study and access an online consent form, complete demographic questions, and provide contact information for interview scheduling. Announcements were also shared with the researchers' social networks to see whether personal contacts knew of potential participants, but this was a far less effective strategy (*n* = 3).

### Interview Procedures

The first author contacted interested participants to arrange a 60‐ to 90‐min semi‐structured interview by telephone (*n* = 18), video call (*n* = 1), or in person (*n* = 1), whichever the participant preferred. The opening question asked participants to share their experience of being separated from their spouse, followed by questions about topics such as the current nature of the marital relationship, reconciliation attempts, and whether or how separation aided in obtaining clarity about the marriage's future. The questions were asked consistently across the interviews. However, because of the semi‐structured nature of the protocol and a desire to follow the participants' lead, the order of the questions varied. Furthermore, one question asked about participants' conceptualizations of separation before separating and how they might have changed because of their own experiences; this question did not generate much by way of responses and as such was not asked in all interviews. Participants were compensated with a $25 gift card to an online retailer. All interviews were audio‐recorded and transcribed verbatim.

### Coding and Analysis

The first author followed procedures for thematic analysis informed by van Manen (1990) and aimed to uncover themes within and across participants' experiences. A theme is an “element (motif, formula or device) which occurs frequently in the text” (van Manen, 1990, p. 78) and an “experiential structure” that makes up part of the experience (p. 79). Collectively, themes provide an interpretation of participants' experiences of a phenomenon and offer descriptions of different experiential structures. First, the researcher read each interview to gain a holistic sense of the text and wrote a summary statement to capture the overall essence of that participant's experience. Second readings helped identify and code what individual sentences or sentence clusters revealed about the separation experience. Each sentence or sentence cluster was assigned a code that captured its essence. Third, each interview was read with the goal of identifying and assigning codes to “key statement(s) or phrase(s) [that] seem particularly essential or revealing” about the experience of being separated from one's spouse (van Manen, 1990, p. 93). Finally, themes were arranged in various levels of abstraction. To determine essential themes, van Manen (1990) suggests asking, “Is this phenomenon still the same if we imaginatively change or delete this theme from the phenomenon?” (p. 107). Those that would substantially change the essence of the phenomenon—namely, those that were most salient across the participants' experiences—were categorized as essential themes. Supporting themes added complexity and depth within the essential themes. Discerning essential from supporting themes was an iterative process that involved several addition immersions in the data; theoretical arrangements were assigned to the data and subsequently revised until the collection of structural experiences captured the similarities across and variations within the participants' experiences.

### Trustworthiness

One of the cornerstones of hermeneutic inquiry is explicating the researchers' “forestructure of understanding” (Chesla, 1995, p. 67). Both authors are licensed marriage and family therapists, have worked extensively with couples considering divorce, and have done research in the area of marital decision‐making. Neither has experienced marital separation personally, but both know others who have. The authors do not assume complete objectivity is possible but aimed to limit the degree to which these experiences influenced the research process and findings. As such, several strategies were employed to enhance trustworthiness according to criteria established by Lincoln and Guba (1985). To increase credibility, the first author met every 1–2 weeks with the second author to discuss the interviews, themes that emerged from the data, and to explicate personal assumptions and biases as they surfaced. To enhance dependability, two external auditors with content and methodological expertise each received two randomly selected, de‐identified, and coded interviews and assessed whether the coding captured the essences of participants' experiences. Their interpretations of the interviews closely aligned with the researchers', and their feedback contributed to more nuanced thoughts about and stronger articulation of the study themes. Lastly, to enhance confirmability, the first author practiced ongoing reflexivity (Lincoln, Lynham, & Guba, 2011) by documenting theoretical ideas and reflections on the participants' narratives and made these memos available to three scholars who were not involved in the study but offered content and methodological expertise.

## Results

All participants were separated at the time of the interviews and cited reasons for separating such as their own or their spouses' desires for clarity about the future of the marriage, time apart to reduce the intensity or frequency of arguments, and to demonstrate the seriousness of their marital complaints. Six essential themes emerged: (a) *Our relationship feels ambiguous*, (b) *separation is a private experience*, (c) *separation is a lonely experience*, (d) *benefits to separating*, (e) *separation is not sustainable*, and (f) *the outcome is unclear*. Descriptions for these themes appear below, along with supporting themes and exemplar participant quotes.

### Essential Theme 1: Our Relationship Feels Ambiguous

One of the most prominent themes was the amount of ambiguity experienced in the relationship with one's spouse, which related to a lack of clarity about the relationship status, uncertainty about new relational boundaries, the degree to which the terms of the separation were clear or shared, and the effects of the separation on family members.

#### Ambiguous status

Unlike marriage and divorce, the participants described not having clarity about the meaning for their separated status, instead using metaphors like “grey space,” “being in limbo,” “sitting on a fence,” “being at a crossroads,” or “living in purgatory,” which implied uncertainty, a sense of waiting, and impermanence in this in‐between status. One woman summarized the lack of clarity she felt this way: “*Are we a couple? Are we a married couple? Are we just…are we just dating? Where to do we stand? What category do we fall into when it comes to, I guess, a couple in general”* (P14).

#### Ambiguous marital boundaries

A lack of clarity about the relationship status resulted in ambiguity in interactions with one's spouse. Almost all participants reported that they or their spouse was more hopeful about reconciliation while the other felt less certain. These dynamics resulted in confusion about the “right” way to interact with each other. Sometimes, boundaries felt unclear because of mixed signals about whether a spouse was leaning toward divorce or reconciliation. Some spoke of times when a spouse displayed behaviors that possibly indicated a desire to reconcile, such as affectionate gestures, requests for time together, or flirtatious messages, while maintaining distance. One man, whose wife had moved out a few months prior, was confused by his wife's behavior: *“Every now and then she was giving the message, or sending me a text message asking me if I'm okay, or telling me she loves me”* (P18). At the same time, many talked about trying to minimize confusion or not send mixed signals while maintaining enough closeness to leave open the possibility for reconciliation. One man described how he felt about spending time with his wife: *“I don't necessarily know where I am…so I worry about giving her a false sense of hope or that things are in a different spot than they are”* (P20). Two spoke of trying to minimize confusion to preserve the integrity of the decision‐making about the marriage's future; being too close with a spouse would distract from the marital problems, which subsequently meant they might not be resolved. Uncertainty about boundaries even extended to public symbols of commitment, such as wearing a wedding ring. One man said about removing his ring: *“I started feeling kind of awkward about [wearing my ring], to the point where when she was around, I was wearing mine, she wasn't wearing hers. [The children] saw me wearing mine and they knew she wasn't wearing hers”* (P10).

#### Separation terms

The marital relationship also felt ambiguous because the separation terms were difficult to negotiate. Given the unclear outcome, some participants did not want to make changes that would be difficult to reverse. One woman said, *“We haven't wanted to do like a cut and dry anything… because I think we both felt like there's just too many unknowns [about our future]”* (P9). Ultimately, the arrangements varied. Some made few financial changes while others became financially independent. Some spent time with their spouse and children together while others only spent time with their children apart from their spouse. However, the negotiation processes for these terms emerged in two forms. A few participants had intentional conversations with their spouse about the terms, usually concerning time with the children, minimizing the impact on the children, and finances. Only five talked about intentional conversations with their spouse about whether they would date other people. However, these arrangements could prove challenging. For example, one man and his wife agreed to see other people. His wife, who initiated the separation, began dating, and he was surprised that she was upset when he decided to go on a date, too: “*She was begrudgingly going out because she was lonely and I wasn't there for her, even though she prefers it was me. Then was upset when I wanted to see somebody else”* (P20).

More often, participants relied on assumptions and beliefs about what separation would mean and did not explicate the terms with their spouse, which led to greater ambiguity. For example, most believed that dating other people would be inappropriate, but few had articulated this with their spouse. One woman said, *“I'm Catholic so to me, that would be a violation of our marriage. Even though we're separated, we are still married people and that's against the rules in my book”* (P12). Some participants even said they would divorce if they learned their spouse had begun a relationship with another person.

#### Familial relationships

The ambiguity of the participants' relationships with their spouses bled into relationships with others, namely their children and extended family. More than those with adult children, participants with school‐aged children cited worry about the effects on their children. Most desired to be honest about the separation, in part because of the potential for their children to feel blindsided by a divorce, while minimizing the impact of the separation on them. Some participants thought their children had adjusted well, but others saw their children as struggling. One woman said, *“We've tried to explain it to her, but then she's like… we just told her that things were best for right now with him staying somewhere else. And I…we needed a break, but she's still not understanding all of that”* (P16).

The participants also described ambiguity in relationships with extended family, who they often perceived as intolerant of or confused by the couple's relationship status. One man said this about his in‐laws: *“The ambiguity is what's bothersome to them. ‘You just need to make a decision!’”* (P11). Other times, this ambiguity related to not knowing how to maintain relationships with those who may not be part of their future. One woman felt confused when her mother‐in‐law had not responded to her attempts to connect: *“I can't figure out why. What have I done to her?...It hurts me thinking that I'm going to lose that relationship, too”* (P16).

### Essential Theme 2: Separation is a Private Experience

This theme captures the degree to which participants made their situations known to others. While a few were more public about their experiences, most shared their experiences with only a small number of confidants and were relatively private about the separation. One woman said it this way: *“To me, it's just kind of private and it's nobody else's business unless I decide to tell them”* (P19). Concerns about others' perceptions and judgment and unhelpful feedback primarily motivated decisions to keep their experiences private.

#### Others' perceptions

Fear of others' perceptions, specifically worries of judgment about having “failed” in the marriage, and becoming fodder for others' gossip, resulted in a great deal of privacy and secrecy surrounding the separation. One woman said it this way: *“I just don't want my bad news to become some gossiper's delight”* (P12). Women specifically feared judgments about not meeting social expectations for wives. One woman described her privacy:

I think women view separation, at least for me, is that I failed somehow at not being able to make this work. That it's a reflection on me as a woman, as a mother, and as a spouse. That I somehow have failed in some way. I failed my husband. I failed my children. And in some ways, I failed myself. (P1)

The participants often struggled with the ambiguity of their situation, so trying to explain it to others felt complicated. One woman talked about not knowing how to talk with her mom: “*She wants to know what's going on, and it's hard to give her answers when I don't know*” (P7). Another woman described attending church without her husband: “*And people kind of look at me weirdly, or ask about [my husband] and I don't really know what to say. Yeah, it's odd*” (P9).

#### Others' responses

While most participants remained private, they had usually confided in a small number of people. Sometimes, they received support and encouragement or help with the children or finances. Most feedback, however, did not acknowledge the complexity of the marital problems, and some participants even felt a need to protect their spouse from undue slander. One man, who initiated the separation, defended his wife: *“I'm more likely to say, ‘Oh, it's me! Please don't blame her because really she's a wonderful person.’ I have my own faults, too”* (P11). Sometimes others' feedback suggested that participants “move on” and date other people. The participants largely rejected these suggestions. One man said, *“I don't think that's real. I don't think that's true. I don't believe that. I don't think I can get over her just like that”* (P17). Often, unhelpful responses reinforced patterns of privacy.

### Essential Theme 3: Separation is a Lonely Experience

This theme captures the participants' feelings of loneliness and isolation as they adjusted to life as separated persons. Their loneliness primarily related to time apart from their spouse and children, daily reminders of the separation and living alone, and feeling burdened by assuming the primary responsibility for children.

#### Time apart

Missing their spouse contributed to participants' feelings of loneliness, sadness, pain, and longing for things to be different. One woman described the tension she felt about her husband's absence: *“The least challenging thing is going to bed alone at night, and the most challenging thing is going to bed alone at night”* (P1). Sometimes loneliness emerged as initial feelings about the marital problems became less intense. One woman, who initiated the separation, said, *“I do miss him. So, [the separation] has kind of provided me with a sense of…I do love him, I want him in my life, and I want to work to make that happen again”* (P12). This woman felt tempted to invite her husband home, even though the marital problems were not resolved. Initial loneliness seemed to fade when clarity about a desire to divorce emerged. One woman now wanted a divorce after separating from a spouse who abused drugs. She compared her initial loneliness with how she later felt:

The first few months were the hardest because you're not sleeping in the same bed as that person. You're not sharing your life the way you were. You're lonely, but now I'm the happiest person in the world. Time healed the wounds, as they say. (P2)

Participants also missed their children, who they saw less because of the separation. One woman felt “lonely too much” when her daughter stayed with her husband (P16). For one man, being apart felt complicated because he initiated the separation: *“It's awful. It's absolutely the worst thing, one of the worst things I've ever experienced. Being separated from my children and my wife…sometimes I just want to be there and be home, but I just can't do that”* (P11).

#### Daily reminders

Reminders of being apart, such as completing tasks the other spouse usually completed or realizations that their spouse was not part of their routines, triggered feelings of loneliness. One man described how these reminders were operative in his emotional experience: *“I could be working feeling normal, I'm feeling alright…and as soon as I start to prepare dinner, I realize I'm cooking for myself only and my emotion can totally change”* (P10).

#### Responsibility for children

In 15 of the 17 cases with minor children, the women assumed primary caretaking responsibilities during the separation. Many were glad to be with their children, but caring for them without their spouse's daily help felt burdensome and lonely. One woman talked about struggling to care for her daughter alone: *“There's not enough hours in a day being a parent to do all these things for a little one. It would just be nice to have another set of hands”* (P4). She then felt shame around other women who appeared to have an easier time parenting alone. Assuming primary responsibility for the children also resulted in less social time, which increased feelings of isolation. This was worse when participants financially supported themselves. One woman felt burdened by working 2–3 jobs to provide for her children: *“Being separated has put a damper on my financial situation, which forces me to work all the time. Me working all the time has put a damper on my social life”* (P13).

### Essential Theme 4: Benefits to Separating

Despite challenges, all but two participants identified some benefit from separating. Specifically, separation took pressure off the relationship, served as an impetus for change, made daily life easier, and spurred realizations about oneself.

#### Pressure off the relationship

For some participants, separation provided a release from the strain of a distressed marriage, resulting in fewer arguments or less intense negative feelings. One woman talked about previously arguing with her husband about not knowing his whereabouts because of his unpredictable work and travel schedule. After separating, she felt less of a need to know where he was, which subsequently resulted in fewer arguments: *“Well, then I'm not waiting for him to get back from like a late flight, you know all those things add anxiety to my day and so it's making it a lot better”* (P3). Interestingly, their sex life also improved after separating. Another participant said he would recommend a separation after frequent arguments diffused when living apart: *“Anybody I know who is kind of in that position, yeah, I would say absolutely yeah. Do a trial separation. Give yourself some space to kind of calm down and get through”* (P11).

#### Impetus for change

For some participants, separating created an impetus for positive change in themselves, their spouse, or their relationship, which provided hope for reconciliation. For example, one man said this about his wife: *“She's now doing individual therapy for herself for the first time, which I guess gives maybe hope and curiosity, like could there be something good that comes out of that that would change the relationship?”* (P20).

#### Easier daily life

Despite the loneliness of being apart, living separately simplified elements of daily life, such as not considering their spouses' preferences, routines, or needs. One woman said, *“I would say that there is a certain ease to independence in some ways like being the full decision maker of the house. It's been kind of nice too, especially because my husband and I are so different”* (P9). One woman felt surprised by this: *“I didn't see him around. I didn't pick up after his mess, I didn't have his mess to pick up after, or like I didn't take him food at work. It was a shock to be honest with you”* (P14). However, this woman realized that her spouse's daily life had become easier since separating. They had separated for 8 months before reconciling; 2 months later, he initiated another separation. She said of his reasons: *“He doesn't have any responsibilities…I take care of the kids all the time. I think he just kind of likes that he doesn't have to think about it”* (P14).

#### Realizations about self

Finally, some participants gained realizations about themselves that felt empowering, as they discovered capacities and resilience they had not previously known. One woman learned of her capacity for independence:

I kind of became reliant on him like we always read a story together, we always did things, like sometimes he'd help me when I couldn't do something and like he would put together stuff and like even today when she had a toy, I was like oh he always puts her toys together… I think it's surprising ‘cause I can do more than I thought I could. (P7)

Sometimes participants' realizations about themselves resulted in more clarity about their desires for the future. One woman talked about the awareness she gained after initiating a separation from her husband who had long abused drugs: “*Doing it all by myself made me feel like, wow I don't need somebody to… you know… I did it by myself”* (P2).

### Essential Theme 5: Separation is Not Sustainable

This theme refers to the notion that separation was an unsustainable marital status. The participants unanimously talked about how they could not go on with separation indefinitely, as the uncertainty of the future was too much to bear, as it resulted in ambiguity and uncertainty in the present. Most indicated a need to decide either way, even if deciding meant divorce; though painful, this seemed preferable to living with ongoing uncertainty. Many participants made comments like this: *“For our own kind of sanity, we would have to separate [permanently] at some point because, you know, I just don't know how I could do that long term”* (P3).

#### Emotional toll of uncertainty

For participants who were deciding about whether to divorce or reconcile, the unsustainability related to the emotional toll they felt about their ambivalence and the effects of needing to decide. When asked what was most challenging, one man talked about not knowing how to decide about the future of his marriage: *“I think some of it is the struggles of figuring out what's next”* (P20). Another woman said, *“Let's just end this because [not knowing is] taking its toll on me emotionally. And I can't handle it much longer”* (P1). For those who wanted reconciliation, waiting on an ambivalent spouse and making meaning for what happened to the marriage were emotionally taxing. One man, whose wife gave little information about why she wanted space, expended considerable energy trying to make sense of her decision. He described his need for an answer this way:

If this goes on for too long it's going to get to the point, I fear, where if I don't have information where I can determine which way we're going…I want to know what the future looks like, and if I can't start seeing which direction it's going, I'm going to start wanting a clear‐cut [decision] one way or the other. (P10)

Most often, the participants' tolerance for ambiguity grew over time. Participants described more intense feelings of sadness, pain, and confusion early on, and they adjusted to living with uncertainty about the marital status and future but only as something to endure for a time. One woman said so concisely, *“I just want it to be over”* (P12).

#### Life is on hold

Many participants described the paralyzing effect of not knowing the separation's outcome and feeling like much of life was on hold until a decision emerged. They felt stuck not knowing how much to adjust their daily lives to accommodate a potentially temporary new normal. One man's example captured this well:

When you separate it goes down to, somebody's going to get the cooking pan. Somebody else is going to have to buy a cooking pan…Then, I was going to go buy a set of pots and pans, and then I actually asked her, “What do I do…what are we going to do about this if I go spend all this money on all of this stuff, and then in 3 months we reconcile everything and you come back, and now we have double of everything?” (P10)

### Essential Theme 6: The Outcome is Unclear

The final theme that emerged was the role and utility of separation in the participants' decision‐making about the future. For some participants, separation helped cultivate clarity; most participants, however, remained unclear about the future of their marriage, were uncertain how to arrive at a clear decision, and worried separation had created momentum toward divorce.

#### Separation brought clarity

Four participants had gained clarity about wanting to divorce; three of these participants had experienced severe marital concerns, such as a spouse's substance use or severe, untreated mental illness, and the fourth decided she did not want to stay with a spouse who had initiated multiple separations. These participants talked about gaining new perspective and not wanting to return to emotionally taxing or unsafe circumstances. One woman said of the verbal and emotional abuse she endured: *“You minimize your own dysfunction. ‘Oh, it's not that bad.’ And then you even get to the point where, ‘I'm the one who caused this, you know. I had too much to drink last night’”* (P18). For this woman, separation and distance from her spouse helped her “get her head on straight.” However, none of these participants had begun divorce proceedings at the time of the interviews, and a couple seemed relatively ambivalent about doing so. One woman cited her hesitance because of limited finances, and another commented on the prospect of an emotionally taxing legal process, for which she did not yet feel ready. None of these participants were clear about when they might begin legal divorce proceedings, but all desired to do so eventually.

#### Separation has not brought clarity

The majority of participants, whose separations began because of less severe reasons such as lack of affection or communication problems, remained unclear about the separation's outcome, either because they were uncertain themselves or because their discerning spouse had not yet arrived at a clear decision. For these participants, separating had not resolved questions about whether to stay married. In response to a question about whether separating had helped her become clearer about the marriage's future, one woman gave what became a common response: *“I don't know. Really, I don't. I mean it's all such a confusing situation”* (P12). Some thought that separating had added confusion and created new challenges to navigate. One woman shared about her unrealized intentions:

I thought [separation] was going to help. I thought this was going to be the answer, that this was going to be just exactly what we needed, and it's not. It's not what we needed. I think that it's making things worse. It's made us grow farther and more distant from each other. (P16)

#### Factors in decision‐making

The participants referred to several factors that influenced their thoughts about the marriage's future. Many considered the effects of their decision on their children. Sometimes, children were a primary reason for considering reconciliation, as described by this man: “*We're just trying to weigh…is it worse for them if we're fighting? Is it worse for them if we're separated?”* (P11). Another woman only considered reconciliation because of her children: “*I would say the most important thing right now is my daughter and her happiness and keeping the family together for her happiness, but, sometimes things don't work out that way, so…”* (P4). Some also considered the dissonance between religious and cultural values and their consideration of divorce. One woman, who separated because of her husband's anger, said,

I'm Catholic so I really don't believe in divorce, but he does believe in divorce. He's Baptist, so it's just really hard for me. I would be really ashamed to have failed at this, but I have to do what's best for me and my children. (P12)

Many also considered finances. Limited financial resources, or the expectation of financial hardship following a divorce, led some participants to more seriously consider reconciliation or delay a formal divorce process. One woman decided she wanted to divorce after her husband initiated a second separation, but she felt conflicted about initiating divorce proceedings because she needed his financial support: *“That's the most difficult thing. I want to make it officially over, but I think that I need his help…And I want to take it as slow as he wants to take it, but I'm like, ‘I'm fed up!’”* (P5). Another woman feared the power her income‐earning spouse would have if he decided to seek full custody of their children: *“I would stay in a miserable marriage for a hundred million years if it meant that my kids wouldn't be taken away from me”* (P12). In contrast, one man talked about the luxury of not worrying about finances: *“We realize we're lucky enough that we can afford to divorce”* (P11). Finally, some wanted evidence of their spouse's changes. One woman initiated a separation after learning about her husband's infidelity. Her decision about whether to stay married primarily rested on him promising that he would not have another affair. She said, *“[We could consider reconciling] if he said that…then we could move forward and stuff”* (P6). Others wondered whether their spouse's changes would last. For example, one man described having sexual problems with his wife that had improved during the separation, but he was unsure if the change would last.

#### Movement toward divorce

Despite openness to reconciliation, several participants lacked confidence they could achieve this and feared that separation had created movement toward divorce, an outcome they were not yet ready to choose intentionally. Others, usually the non‐initiators, wanted to reconcile but feared that divorce was becoming inevitable as they waited for an uncertain spouse to decide. Some described this movement as a function of time; the longer they stayed separated, the harder it would be to reconcile. One woman hypothesized about how long she could continue in a separation before making a final decision: *“I think I'll know when, if we're in the same place we are 6 months from now, then it's time to move on. It's time to either shit – excuse my French – shit or get off the pot”* (P1). Another woman said, *“Days turn into months and…nothing's changing and you kind of feel like it's gonna go there, but you really don't want it to. If it doesn't change, I do think it could, because I can't live that way”* (P7). Others saw this movement as a function of adjusting to life apart. One woman wondered if her husband had gotten too used to being alone to consider reconciling: *“You start developing other habits of doing things on your own, and you become independent. And I think that's what he liked about it, is I guess just not being tied down”* (P14). One woman talked about her fear of divorce as she and her husband grew distant and spent less time together: *“As much as I don't want it, I think it's probably leading toward divorce. I don't know, the further we grow apart the more risky…the longer, too, and the more risky…it's like, what is going to be next?”* (P16). Lastly, some participants saw this movement as a function of unclear solutions to the marital problems. One woman talked about her concern that she would divorce because she and her husband were at a loss for how to resolve the problems that preceded the separation*: “And it's very tough because, I probably sound like a broken record, but we honestly want to try to figure it out. And I feel like there's no blueprint for us.”* (P1).

## Discussion

The purpose of this study was to investigate the under‐studied experience of ambiguous marital separation. There seems to be a bias in the literature toward the inevitability of divorce following separation, as reflected by the ways separated persons are conflated with divorcing persons in study samples (e.g., Sbarra, Smith, & Mehl, 2014). However, the experiences of this study's participants suggest that separation does not always begin with intentions to divorce. Decision‐making about the future of a marriage seems to extend beyond a decision to separate, which is consistent with research on ambivalence related to divorce decisions (Doherty, Willoughby, & Peterson, 2011). Interest in marital and divorce decision‐making processes is growing (e.g., Allen & Hawkins, 2017; Hawkins et al., 2017), and researchers may benefit from examining these processes across a variety of marital arrangements, statuses, and transitions.

One of the most central themes of the participants' experiences was the level of ambiguity surrounding separation. The participants struggled to attribute meaning to their marital status, and they described varying levels of uncertainty about how to interact with their spouse or negotiate the terms of the separation. This theme resonates with Boss and Greenberg's (1984) concept of family boundary ambiguity, referring to a lack of clarity about the permanence of a relational loss. While structural changes to family life contribute to feelings of uncertainty, perception of and meaning for these changes are most predictive of experiencing boundary ambiguity (Carroll, Olson, & Buckmiller, 2007), and higher levels and prolonged periods of boundary ambiguity result in increased levels of stress (Boss & Greenberg, 1984). The participants almost unanimously spoke of separation as unsustainable, and some hypothesized that a clear decision to divorce might provide relief from distress associated with ambiguity, even when divorce was not the preferred outcome. Previous research has documented the detrimental mental health effects of intolerance of uncertainty (e.g., Carleton et al., 2012). While boundary ambiguity has been observed among some divorcees as well (Peterson & Hendricksen Christiansen, 2008), the prospect of clarity in the midst of an ambiguous experience might motivate decisions to pursue divorce. Future research could investigate whether boundary ambiguity and intolerance of uncertainty account for any ambivalence among divorcing persons (Doherty, Willoughby, & Peterson, 2011). Researchers could also examine whether articulated relational boundaries and separation terms influence decision‐making. Violations of unspoken boundaries, such as dating other people, may add new challenges to consider.

Some participants also described concerns that separation created unwanted movement toward divorce. Stanley and Markman's (1992) commitment model examines marital outcomes based on whether relationships progress as a function of prosocial commitment to the relationship or constraint commitment—staying because the constraints are too great to leave. They refer to the latter as an “inertia effect,” which results in couples “sliding versus deciding” about relationship progression because leaving becomes more difficult as a couple becomes increasingly interdependent (Stanley, Rhoades, & Markman, 2006, pp. 503–504). Similarly, separation may create movement—or inertia—toward divorce because constraints to staying married seemingly decrease as spouses' lives become less entangled. Some participants talked about the perceived unlikelihood of reconciliation as a function of time or adjusting to life apart, or as mentioned previously, the intolerable nature of ongoing ambiguity. Instead of cultivating confidence about a decision to divorce, separation may lead some couples to slide into divorce but with some ambivalence. Stanley and Markman's (1992) commitment model may also help make sense of reasons some couples reconcile and the dissatisfaction and instability of reconciled marriages following separation (Binstock & Thornton, 2003; Kitson, 1985; Vennum et al., 2014; Wineberg & McCarthy, 1994). While most participants described having some desire for reconciliation, some felt inclined to stay married because of other constraints, such as financial strain or the expectation of financial hardship following a divorce. This seemed especially prevalent among the women. While this is curious given the sample's reported affluence, family stress, and coping researchers (e.g., Boss, 1992) hold that the perception of resources is often more pertinent in stress responses than the actual presence of resources. Furthermore, women are more likely to endure financial hardship following divorce (de Vaus et al., 2017), and previous separation research supports the notion that those with fewer financial and social resources are more likely to stay married or attempt reconciliation (Morgan, 1988; Tumin et al., 2015; Wineberg & McCarthy, 1994). It may be that the perception of constraints or expectation of hardship, financial or otherwise, motivates decisions to stay married. Future research could investigate whether trajectories of couples that reconcile because of constraint commitment differ from those who reconcile because of prosocial relationship commitment. The latter may be more inclined to work on the problems that preceded a separation, thereby reducing subsequent marital instability compared with couples that reconcile because of constraints.

The participants also described keeping separation relatively private out of concern for others' perceptions and receiving unhelpful feedback. The women seemed particularly worried about not meeting social expectations or that others would see their struggling marriage as a reflection of their inability to keep their relationship healthy, which is consistent with previous research showing that women tend to feel more responsible for maintaining a relationship's health (Baum, 2007). The participants' concerns about others' feedback is also consistent with previous findings about the helpfulness of confidants' responses. People who have confided in others about their marital or relationship problems have reported the least helpful responses as giving too much or unhelpful advice, talking too much about oneself, being too critical of the confider's spouse or partner, being too judgmental or critical, and suggesting the confider end the relationship (Lind Seal, Doherty, & Harris, 2016). Together, these nuances of privacy suggest there appears to be lasting stigma around marital problems, some of which may be disproportionately gendered, despite evidence that Americans are increasingly accepting of divorce (Dugan, 2017). However, the participants' privacy also raises questions about the prevalence of ambiguous separation, which is difficult to track because separation does not necessarily involve a legal change in marital status.

Finally, the study themes call into question the utility of separation for deciding whether to stay married as promoted by popular sources (e.g., Lipe, 2010; Raffel, 1999). Persons considering divorce desire but often struggle to obtain clarity and confidence about a marriage's future (Harris, Crabtree, Bell, Allen, & Roberts, 2017), and based on the participants' experiences, separation did not usually provide such clarity. Some even reported that separating added to their confusion. However, four participants had arrived at desires for divorce, and three of these cited severe problems such as their spouse's substance abuse or untreated mental health conditions as central in their decision‐making. Those who separated for less severe reasons were generally less clear about what they wanted, or desires for reconciliation accompanied uncertainty about solutions. While our sample size and methodology do not lend to generalizability, these patterns might warrant future attention to the relationships between marital problems and the utility of separation for marital decision‐making.

### Clinical Implications

Therapists are uniquely positioned to help persons uncertain about the future of their marriages achieve clarity in their marital decision‐making, and these findings may provide guidance for this work. First, more research is needed to determine when or if separation is indicated, and therapists should be cautious about encouraging separation for the purpose of marital decision‐making until this is more thoroughly understood. Second, therapists should be mindful that uncertainty might continue beyond a decision to separate. If therapists share the assumption that separation inevitably leads to divorce, they may prematurely encourage divorce and dissolution processes. Holding space for uncertainty may help preserve the integrity of a couple's decision‐making. Lastly, if a couple separates, therapists may be able to help them articulate a plan that mitigates movement toward an undesirable outcome. Couples may benefit from explicating clearer boundaries to reduce ambiguity and purposefully negotiate terms that might otherwise disproportionately affect one spouse, such as childrearing responsibilities.

### Limitations

This study is not without limitations. Our sample was relatively homogenous, which may be the result of recruiting participants through social media advertisements. Over 75% of the participants identified as white, and 70% identified as women. Different themes may emerge with samples that are more diverse in race and gender, or who do not use the social media platform we utilized for recruitment. Furthermore, all participants were in female–male marriages. We did not intentionally exclude participants in same‐sex marriages but were unsuccessful in locating any for this study. In addition to the chosen recruitment strategy, this may be a function of the criteria that participants be married and the relatively little time same‐sex couples have been able to legally marry. Furthermore, recent evidence suggests that same‐sex divorcees may worry their experiences render them illegible because of heteronormative assumptions (Hoy, 2018), and it may be that separated persons in same‐sex marriages feared additional stigma. There are challenges related to reaching hidden populations (Ellard‐Gray, Jeffrey, Choubak, & Crann, 2015), and the participants' notion that separation is a private experience points to the hidden nature of separated persons more broadly. Researchers should consider this in future attempts to locate study participants, especially those who belong to marginalized populations. The cross‐sectional nature of the data is also a limitation. Most participants indicated that separation had not helped them gain clarity about the marriage's future, but the outcomes of their separations are ultimately unknown. Collecting data across multiple time points may illuminate how ambiguous separations and related decision‐making processes may progress and are experienced across time.

### Directions for Future Research

Several possibilities for advancing the literature on marital separation emerged from this study. First, acquiring data on the prevalence of ambiguous separations may be useful, as the percentage of separations that begin without clarity about how they will end is unknown. Separation and divorce researchers should ask about separation intent, even of divorcing or divorced persons, as this could help provide insight into the varying pathways couples take in arriving at decisions to divorce. Those who begin with an ambiguous separation and later divorce could report on about how they arrived at their decision, which could shed light on thresholds for ambiguity, lack of clarity about how to solve marital problems, or other factors that led to their decision, such as unclear relational boundaries. It may be that some couples actually “slide” into divorce without confidence that divorce is truly the best option (Stanley, Rhoades, & Markman, 2006, p. 505), and movement created by separation could help account for some of their ambivalence about divorce. This research could also benefit from more diverse samples, including participants in same‐sex marriages. There may also be value in investigating whether ambiguous separation is experienced differently in unmarried cohabiting couples who perhaps do not have the same legal constraints to divorce but have made similar levels of relationship commitment. Finally, given the popular notion that separations are helpful for decision‐making and the potential role of a therapist in helping couples discern whether to stay married, assessing therapist attitudes about ambiguous separations may be important.
